# Impact of Midline Rectovaginal Fascia Plication on Female Sexual Function and Surgical Outcomes: A Retrospective Study

**DOI:** 10.3390/jcm15135173

**Published:** 2026-07-02

**Authors:** Asiye Uzun, İsmail Bıyık, Zehra Yılmaz, Güneş Topçu, Ümit Zeteroğlu, Şafak Hatırnaz, Radmila Sparić, Andrea Tinelli

**Affiliations:** 1 Department of Obstetrics and Gynecology, Bahcelievler Medipol University Hospital, Istanbul Medipol University, 34214 Istanbul, Turkey; asiye.uzun@medipol.com.tr (A.U.); drguneskoc@gmail.com (G.T.); 2 Department of Obstetrics and Gynecology, School of Medicine, Kutahya Health Sciences University, 43100 Kutahya, Turkey; dribiyik@hotmail.com; 3 Dr. Zehra Yilmaz Clinic, Alacam Caddesi, 55200 Samsun, Turkey; 4 Ozel Mozaik Kadin Hastaliklari ve Dogum Hastanesi, 31070 Hatay, Turkey; umitzeteroglu@gmail.com; 5 ES Hatırnaz Clinic, Atakum, 55200 Samsun, Turkey; safakhatirnaz@gmail.com; 6 Clinic of Gynecology and Obstetrics, University Clinical Center of Serbia, Faculty of Medicine, University of Belgrade, 11000 Belgrade, Serbia; radmila@rcub.bg.a.c.rs; 7 Department of Obstetrics and Gynecology, and CERICSAL (CEntro di RIcerca Clinico SALentino), “Veris Delli Ponti Hospital”, Via Giuseppina Delli Ponti, 73020 Scorrano, LE, Italy; andreatinelli@gmail.com

**Keywords:** rectocele, midline rectovaginal fascia plication, female sexual function, female sexual function index (FSFI), vaginal relaxation

## Abstract

**Objective:** Rectocele is a common posterior compartment defect that may cause defecatory dysfunction and impaired sexual function, adversely affecting quality of life. Although midline rectovaginal fascia plication (MFP) is associated with favorable anatomical outcomes, its effects on female sexual function remain insufficiently explored. This study aimed to evaluate surgical outcomes and changes in female sexual function following MFP in women with symptomatic rectocele. **Methods:** This retrospective study included 100 women who underwent MFP for symptomatic rectocele between January 2023 and December 2024. Objective and subjective success rates were assessed using POP-Q findings and symptom resolution. Sexual function was evaluated preoperatively and at 12 months postoperatively using the Female Sexual Function Index (FSFI). Postoperative pain was assessed using the Visual Analog Scale (VAS). **Results:** The mean age was 45.96 ± 10.12 years, and the mean follow-up period was 14.42 ± 3.57 months. Objective success based on POP-Q findings and subjective success based on symptom improvement were 92% and 89%, respectively. Postoperative pain decreased significantly within the first postoperative week (*p* < 0.001). Significant improvement was observed across all FSFI domains. The mean total FSFI score increased from 10.40 ± 4.26 preoperatively to 23.60 ± 3.46 postoperatively (*p* < 0.0001). **Conclusions:** Midline rectovaginal fascia plication is a safe and effective surgical option for symptomatic rectocele, providing high anatomical success and significant improvements in female sexual function, with minimal morbidity and rapid recovery.

## 1. Introduction

While the true incidence of rectocele is not well defined, asymptomatic posterior compartment prolapse may be present in about 40% of women with a history of vaginal delivery [1]. Risk factors for rectocele are increasing age, multiparity, prior pelvic surgery, and disorders associated with chronically elevated intra-abdominal pressure [1]. Rectocele is associated with pelvic pressure or pain, sexual dysfunction, particularly dyspareunia, and defecatory disturbances such as obstructed defecation, incomplete evacuation, and flatus or fecal incontinence [2]. Rectocele is linked to impaired quality of life and has a negative impact on sexual health [3,4].

Surgical management options include posterior colporrhaphy using native vaginal tissue, midline rectovaginal fascial plication, site-specific defect repair, posterior vaginal mesh placement, transanal repair, transperineal repair, and abdominal procedures performed through open, laparoscopic, or robotic techniques [4]. Although the use of mesh may provide stronger tissue reinforcement, transvaginal mesh products for pelvic organ prolapse repair have been withdrawn from the market by the U.S. Food and Drug Administration, which has significantly limited their role in rectocele surgery [5]. Although both transvaginal and transanal techniques improve posterior compartment defects and quality of life, the transvaginal approach seems to achieve higher anatomical success with reduced postoperative pain and fewer complications compared with stapled transanal rectal resection [6]. Midline rectovaginal fascia plication (MFP) is a surgical technique associated with high anatomical and functional success rates, low complication rates, and high patient satisfaction in vaginal reconstruction [7,8].

Rectocele repair is well known to improve women’s sexual function, providing both anatomical and functional benefits [9,10]. Among the available surgical approaches, midline rectovaginal fascia plication (MFP) stands out for its high success rates, low complication rates, and positive patient-reported outcomes [7]. Despite these advantages, the specific impact of MFP on sexual health has not been thoroughly investigated, leaving a gap in the literature. This study aimed to address this gap by evaluating the surgical and clinical outcomes of MFP, as well as its effects on sexual health.

## 2. Materials and Methods

This retrospective study included patients who underwent surgery between January 2023 and December 2024. Ethical approval for retrospective data collection and analysis was obtained from the local Ethics Committee on 25 December 2025 (decision number: 1643). The study was conducted in accordance with the principles of the Declaration of Helsinki.

This retrospective study was conducted with 100 women aged between 29 and 65 years who underwent surgical treatment for rectocele at the Department of Obstetrics and Gynecology, Medipol University, Bahçelievler Hospital. All procedures were performed within the same clinical setting by the same experienced gynecologic surgeon (AU).

Patients who presented with complaints of vaginal laxity and were deemed suitable for surgical intervention were included in the study. Demographic characteristics, obstetric history, surgical data, and postoperative outcomes were obtained from hospital medical records. All patients provided informed consent prior to surgery. Preoperative and postoperative Female Sexual Function Index (FSFI) data were routinely collected as part of standard clinical follow-up and analyzed retrospectively.

Adult women with at least one prior vaginal delivery were eligible for the study. Patients were required to have posterior compartment prolapse stage ≥ II according to the POP-Q classification and a diagnosis of symptomatic rectocele. Eligible patients reported posterior compartment–related symptoms, such as a sensation of vaginal laxity, vaginal fullness, or difficulty with defecation.

Patients were excluded if they had advanced pelvic organ prolapse involving compartments other than the posterior wall, marked perineal distortion requiring additional reconstructive procedures, unexplained uterine bleeding, ongoing pregnancy, known or suspected gynecologic malignancy, collagen diseases, insulin-dependent diabetes or a history of pelvic irradiation. Women with conditions that could independently affect sexual function or pelvic floor integrity—such as deep infiltrating endometriosis, vaginismus, or primary orgasmic dysfunction—were a priori excluded. In addition, cases in which the sexual partner had a diagnosed sexual dysfunction were excluded from the study to minimize confounding factors in postoperative functional assessment.

Postmenopausal patients received topical vaginal estriol cream (Estriol^®^ 1 mg/g; Assos Pharmaceuticals, İstanbul, Türkiye) once daily for 60 days prior to surgery and were advised to continue treatment throughout the follow-up period. Mechanical thromboprophylaxis with graduated compression stockings was applied to all patients. In those considered at high risk for venous thromboembolism, additional pharmacologic prophylaxis with low-molecular-weight heparin was administered. A single dose of 1 g intravenous cefazolin was given approximately 30 min before the surgical incision for antibiotic prophylaxis. All patients underwent preoperative bowel preparation with an enema prior to surgery.

The procedures were performed under general anesthesia in the dorsolithotomy position following vaginal antiseptic preparation with povidone–iodine. The posterior lip of the cervix was grasped with a single-tooth tenaculum. Hydrodissection was carried out carefully between the vaginal mucosa and the underlying fascia without entering the fascial layer.

A linear incision was initiated just anterior to the hymenal ring and extended posteriorly toward the cervix while maintaining traction on the cervix. The vaginal mucosa was dissected off the underlying fascia, and the dissection was laterally extended to the pelvic sidewalls, preserving the rectovaginal fascia. Posterior to the cervix, dissection was continued bilaterally until the uterosacral ligaments were identified, and each ligament was secured with forceps. During bimanual examination, a finger was introduced into the rectum to delineate the fascial defect. The apex of the defect was grasped with two Allis clamps. Using 0 polyglactin sutures, a plication was performed beginning from the left uterosacral ligament to the left rectovaginal fascia, then continued to the right rectovaginal fascia and right uterosacral ligament, with the sutures tied to achieve midline reinforcement. Subsequently, the rectovaginal fascia was further plicated in a continuous manner down to the level of the fossa navicularis using 2-0 polyglactin (Doğsan Medical, Trabzon, Türkiye) sutures. Excess vaginal mucosa was excised, and the vaginal epithelium was closed with 2-0 rapidly absorbable polyglactin sutures.

Patients were evaluated postoperatively at 1 week, 6 weeks, 6 months, and 12 months. Follow-up assessments were conducted during outpatient visits and included a standardized gynecological examination with POP-Q measurements, as well as evaluation of patient-reported symptoms.

Surgical success was determined based on findings at the 12-month follow-up visit. Sexual function was assessed using the FSFI questionnaire, which was administered preoperatively and repeated at the 12-month postoperative visit. All questionnaires were completed during face-to-face clinical evaluations conducted by the same clinician, ensuring standardized administration, patient privacy, and consistency of data collection.

Objective success was defined as the absence of posterior compartment prolapse stage II or higher at the 12-month follow-up according to the POP-Q classification. Recurrence was defined as posterior compartment prolapse reaching stage II or higher during follow-up. Subjective success was assessed based on the resolution or marked improvement of patients’ symptoms.

Improvement in sexual function, as measured by changes in FSFI scores, was evaluated as a secondary outcome. Sexual function was evaluated using the Turkish version of the FSFI, originally developed by Rosen et al. [11] and validated in Turkish by Aygin and Aslan [12]. The FSFI includes six domains: desire, arousal, lubrication, orgasm, satisfaction, and pain, as well as a total score. The FSFI questionnaire was administered preoperatively before the initiation of any hormonal treatment and repeated at 12 months postoperatively during follow-up visits. All questionnaires were completed during face-to-face clinical evaluation. FSFI domain scores were calculated according to the standard scoring system, and the pain domain was recoded so that higher scores indicate less pain. Patients with incomplete preoperative or postoperative FSFI questionnaires were excluded from the final analysis to ensure the accuracy and completeness of sexual function assessment.

Early mobilization and initiation of oral intake were encouraged 6 h after surgery. Patients were typically discharged on the first postoperative day. Follow-up assessments were performed at 1 week, 6 weeks, 6 months and 12 months. For the assessment of surgical success, the 12-month follow-up data were used for analysis. At each visit, patients were evaluated for potential complications such as infection, hematoma formation, and vaginal stenosis. Sexual activity was permitted after 6 weeks.

Statistical analyses were performed using IBM SPSS Statistics, Version 27.0 (IBM Corp., Armonk, NY, USA). The distribution of continuous variables was assessed for normality prior to analysis. Categorical variables were expressed as numbers and percentages (*n*, %), while continuous variables were presented as mean ± standard deviation (SD). Preoperative and postoperative FSFI scores were compared using the paired *t*-test. Additional exploratory analyses were performed to evaluate the association between improvement in total FSFI score and age, BMI, smoking status, menopausal status, and chronic disease history. Pearson correlation analysis was used for continuous variables, and point-biserial correlation analysis was used for dichotomous variables. A *p* value < 0.05 was considered statistically significant.

## 3. Results

### 3.1. Demographic and Surgical Characteristics

A total of 100 patients were included in the study. The demographic and surgical characteristics of the study population are summarized in Table 1. The mean age was 45.96 ± 10.12 years, and the mean body mass index was 27.55 ± 2.72 kg/m^2^, indicating a predominantly overweight study population. The mean operation duration was 57.71 ± 5.39 min. The mean follow-up period was 14.42 ± 3.57 months.

The mean gravidity was 4.17 ± 1.72, and the mean parity was 3.54 ± 1.55. Regarding educational status, 47% of the patients had completed university education, while 27% were high school graduates. Smoking was reported by 51% of the patients.

Most patients (71%) had no significant comorbidities. Among those with comorbidities, hypertension and diet-controlled diabetes were the most common conditions. The majority of patients were premenopausal (78%), and 10% had vaginal atrophy. The mean VAS score at 24 h postoperatively was 3.44 ± 1.14, which significantly decreased to 1.86 ± 1.01 at 1 week after surgery (*p* < 0.001), indicating a rapid reduction in postoperative pain. No major intraoperative or postoperative complications were observed during the follow-up period. Specifically, no cases of postoperative infection, hematoma formation, vaginal stenosis, de novo dyspareunia, or reoperation were recorded.

### 3.2. Primary Outcomes

The objective success rate, defined as the absence of posterior compartment prolapse stage II or higher at 12 months, was 92% (92/100). Subjective success, defined as resolution or marked improvement of symptoms, was achieved in 89% (89/100) of patients. Recurrence occurred in 8 patients (8%), and no patient required reoperation during the follow-up period.

### 3.3. Secondary Outcomes

Changes in sexual function following surgery are presented in Table 2. A comparison of preoperative and postoperative FSFI scores demonstrated a statistically significant improvement in all domains. The mean total FSFI score increased from 10.40 ± 4.26 preoperatively to 23.60 ± 3.46 postoperatively (*p* < 0.0001).

Significant improvements were observed in desire, arousal, lubrication, orgasm, and satisfaction domains (all *p* < 0.0001). Additionally, the pain domain score significantly improved postoperatively, indicating a reduction in dyspareunia-related symptoms. Exploratory analyses demonstrated no significant association between improvement in total FSFI score and age (r = 0.020, *p* = 0.846), BMI (r = −0.008, *p* = 0.936), smoking status (r = 0.182, *p* = 0.070), menopausal status (r = −0.026, *p* = 0.797), or chronic disease history (r = 0.106, *p* = 0.294).

## 4. Discussion

In this study, midline rectovaginal fascia plication (MFP) demonstrated excellent surgical and clinical outcomes. The procedure was performed in a cohort of 100 patients with a mean age of 46 years and a relatively short mean operative time of approximately 58 min. Most patients had no significant comorbidities, and the majority were premenopausal. Both objective and subjective success rates were high (92% and 89%, respectively), and no reoperations were required during the follow-up period. Postoperative pain was mild and showed a rapid decline within the first postoperative week. Importantly, sexual function significantly improved across all FSFI domains, including desire, arousal, lubrication, orgasm, satisfaction, and pain, indicating meaningful functional and quality-of-life benefits. Taken together, these findings suggest that MFP is an effective and safe technique that provides durable anatomical correction while also positively influencing sexual health, with minimal morbidity and rapid recovery. The magnitude of improvement observed in our cohort, with the mean FSFI score increasing from 10.40 to 23.60, further supports the substantial functional benefit of this technique.

The results of the present study are consistent with previously published data in the literature. In a prospective study by Bedel et al. (2022), anatomical success was achieved in 28 of 30 patients (93%) undergoing MFP, with outcomes assessed using POP-Q measurements [8]. The authors reported a mean follow-up period of 12 months and highlighted high anatomical success rates accompanied by low morbidity. These findings closely parallel our results and further support the effectiveness and safety of MFP in the surgical management of rectocele. Maher et al. evaluated the efficacy of midline rectovaginal fascial plication in women with symptomatic rectoceles and obstructed defecation in a prospective study including 38 patients [7]. High subjective and objective success rates were reported at both 12 and 24 months of follow-up. Significant improvements were observed in POP-Q measurements and rectocele depth, along with marked functional benefits, with the majority of patients experiencing resolution of obstructed defecation. In addition, anatomical correction was associated with improvements in dyspareunia and very high patient satisfaction rates. These findings are consistent with our results and underscore the effectiveness of MFP in achieving both anatomical correction and functional improvement.

Site-specific fascial repair has been reported to achieve higher success rates than traditional colporrhaphy in rectocele repair and vaginoplasty [13]. Although direct comparative studies between site-specific fascial repair techniques and midline rectovaginal fascia plication (MFP) are lacking, available evidence suggests that MFP is associated with favorable patient satisfaction outcomes [7,8]. Singh et al. (2003) [14] reported in a prospective study of 42 patients with isolated symptomatic rectocele treated with midline rectovaginal fascial plication an anatomical success rate of 92% at 18 months. Improvements were observed in vaginal protrusion (92%), defecatory function (81%), and sexual function (35%) [14]. Posterior vaginal wall reconstruction not only addresses defecation-related symptoms but also contributes to the improvement of sexual function [4]. In line with the existing literature, our study demonstrated high objective and subjective success rates following MFP, reaching 92% and 89%, respectively. These findings reinforce the effectiveness of this technique not only in achieving durable anatomical correction but also in meeting patient-reported expectations. Beyond anatomical outcomes, a significant and consistent improvement was observed across all domains of sexual function. Postoperative FSFI scores showed marked improvement in desire, arousal, lubrication, orgasm, satisfaction, and pain, highlighting the positive impact of posterior vaginal wall reconstruction on overall sexual health. Taken together, these results support the concept that successful posterior vaginal wall reconstruction addresses defecation-related symptoms while also providing meaningful improvements in sexual function and quality of life. Bedel et al. assessed sexual function using the PISQ-12 questionnaire and reported no significant differences between preoperative and postoperative scores at 12 months following MFP [8]. In contrast, in our study, sexual function was evaluated using the FSFI, and a significant improvement was observed across all domains at one year postoperatively. This discrepancy may be explained by differences in the assessment tools used. Although postoperative FSFI scores improved significantly across all domains, they remained below the validated cutoff value of 26.55 for normal sexual function, suggesting partial rather than complete functional recovery. This finding highlights that while anatomical correction contributes to functional improvement, additional factors influencing sexual function may persist. Factors such as smoking, menopausal status, and hormonal therapy may have influenced sexual function outcomes and should be considered when interpreting the results. While PISQ-12 is designed specifically for women with pelvic floor disorders and focuses primarily on symptom-related sexual concerns, FSFI provides a more comprehensive and sensitive evaluation of female sexual function across multiple domains. Additionally, the larger sample size in our study may have increased the statistical power to detect meaningful changes. These findings suggest that improvements in sexual function following MFP may be more readily detected when assessed using detailed and domain-specific instruments such as the FSFI.

In the present study, exploratory analyses demonstrated no significant associations between postoperative improvement in sexual function and age, BMI, smoking status, menopausal status, or chronic disease history. Although sexual function is influenced by multiple biological and psychosocial factors, these findings suggest that the observed improvements in FSFI scores following MFP were not substantially affected by the evaluated demographic and clinical characteristics within our cohort.

Brandner et al. evaluated sexual function following posterior fascial repair for symptomatic rectoceles using the FSFI and reported significant improvements in desire, satisfaction, and pain domains, while no significant changes were observed in arousal, lubrication, or orgasm [9]. In contrast, our study demonstrated significant postoperative improvement across all FSFI domains at one-year follow-up. Several factors may explain this difference. First, the study population in Brandner et al. was considerably older, with a median age of 72 years, whereas the majority of our patients were premenopausal, which may have influenced sexual responsiveness and recovery potential. Second, sexual function in their study was assessed at 6 months postoperatively, while our evaluation at 12 months may have allowed sufficient time for tissue healing and functional adaptation. Finally, the use of a standardized midline rectovaginal fascia plication technique in our cohort may have provided more consistent anatomical support, potentially contributing to broader improvements in sexual function. Hoda et al. prospectively evaluated sexual function following vaginal surgery for pelvic organ prolapse using transobturator mesh implants and demonstrated a significant improvement in overall FSFI scores during long-term follow-up [15]. Although rectocele patients were included in their cohort, outcomes were reported for a heterogeneous pelvic organ prolapse population without compartment-specific analysis. In addition, an initial decline in sexual function was observed during the early postoperative period, followed by gradual improvement after three months, likely reflecting the healing process associated with the presence of prosthetic material. In contrast, our study focused exclusively on posterior compartment repair using midline rectovaginal fascia plication (MFP) with native tissue, allowing a more targeted evaluation of sexual function. In our cohort, significant improvement was observed across all FSFI domains at one year postoperatively, without an early postoperative deterioration. These differences suggest that compartment-specific native tissue reconstruction may provide earlier and more consistent improvements in sexual function compared to mesh-based techniques.

Female sexual function is a multifactorial outcome influenced by biological, hormonal, psychological, relational, and sociocultural factors. In addition, women with more severe pelvic floor symptoms may be less likely to remain sexually active, which should be considered when interpreting postoperative changes in FSFI scores. Furthermore, sexual satisfaction may not be solely determined by anatomical correction and can be influenced by multiple individual and partner-related factors. Therefore, the observed improvements in sexual function should be interpreted within the broader context of factors that may influence sexual health.

Taken together, the findings of the present study and the available literature suggest that posterior vaginal wall reconstruction plays a crucial role not only in correcting defecation-related symptoms but also in improving sexual function. Our results demonstrate that midline rectovaginal fascia plication using native tissue provides high objective and subjective success rates, minimal morbidity, and rapid postoperative recovery, accompanied by significant and consistent improvements across all domains of sexual function. When compared with previously published studies, differences in patient selection, surgical technique, use of synthetic materials, and assessment tools appear to substantially influence sexual function outcomes. In this context, compartment-specific native tissue repair using MFP emerges as a reliable and effective approach for the management of symptomatic rectocele, offering meaningful anatomical correction while also enhancing patient-reported sexual health and overall quality of life.

### Strengths and Limitations

The strengths of this study include its relatively large sample size and the use of a standardized surgical technique focused exclusively on posterior compartment repair. Sexual function was systematically assessed using the validated Female Sexual Function Index, allowing a comprehensive evaluation across multiple domains rather than symptom-based measures alone. In addition, both objective and subjective success rates were reported, providing a balanced assessment of anatomical and patient-reported outcomes.

Several limitations should be acknowledged. The retrospective nature of the study and the absence of a control or comparison group, such as traditional posterior colporrhaphy or alternative repair techniques, limit the ability to draw causal inferences. The study was conducted at a single center, which may affect the generalizability of the findings. Although sexual function was evaluated at one year postoperatively, longer-term follow-up would be valuable to assess the durability of both anatomical and functional outcomes. Finally, vaginal atrophy, estriol treatment, partner-related factors, and other psychosocial variables that may influence sexual function were not specifically analyzed. Although exploratory analyses were performed for age, BMI, smoking status, menopausal status, and chronic disease history, comprehensive multivariable modeling was not conducted because of the retrospective study design.

## 5. Conclusions

Midline rectovaginal fascia plication was associated with high anatomical and clinical success rates, minimal morbidity, rapid recovery, and significant improvements in sexual function among women with symptomatic rectocele. Improvements were observed across all FSFI domains, suggesting potential benefits beyond anatomical correction alone. These findings support the potential role of compartment-specific native tissue posterior vaginal wall reconstruction in improving functional and quality-of-life outcomes. However, prospective controlled studies with longer follow-up are warranted to confirm these findings and further evaluate factors influencing postoperative sexual function.

## Figures and Tables

**Table 1:** Demographic and surgical characteristics of the patients (*n* = 100).

Age (years)	45.96 ± 10.12	
BMI (kg/m^2^)	27.55 ± 2.72	
Operation duration (min)	57.71 ± 5.39	
Follow-up (months)	14.42 ± 3.57	
Gravidity	4.17 ± 1.72	
Parity	3.54 ± 1.55	
Vaginal delivery	3.08 ± 1.44	
Educational status	Illiterate	19 (19%)
	Primary school	7 (7%)
	High school	27 (27%)
	Graduate	47 (47%)
Smoke	No	49 (49%)
	Yes	51 (51%)
Chronic disease	No chronic disease	71 (71%)
	hypertension	11 (11%)
	diabetes mellitus	11 (11%)
	asthma	5 (5%)
	coronary artery disease	2 (2%)
Menopausal status	Premenopausal	78 (78%)
	Postmenopausal	22 (22%)
Vaginal atrophy	No	90 (90%)
	Yes	10 (10%)
VAS score at 24 h	3.44 ± 1.14	
VAS score at 1 week	1.86 ± 1.01	

Data are presented as numbers and percentages (*n*/%) for categorical variables and mean ± standard deviation (SD) for continuous variables. BMI: body mass index.

**Table 2:** Comparison of preoperative and postoperative FSFI scores (*n* = 100).

	Preoperative	Postoperative	*p*
Desire	2.20 ± 0.82	3.83 ± 0.84	<0.0001
Arousal	1.90 ± 1.14	3.78 ± 0.69	<0.0001
Lubrication	1.70 ± 1.12	3.68 ± 0.80	<0.0001
Orgasm	1.61 ± 1.15	4.21 ± 0.74	<0.0001
Satisfaction	2.00 ± 0.79	4.29 ± 0.83	<0.0001
Pain	0.99 ± 0.75	3.81 ± 0.91	<0.0001
Total score	10.40 ± 4.26	23.60 ± 3.46	<0.0001

Data are presented as mean ± standard deviation (SD). Preoperative and postoperative FSFI scores were compared using the paired *t*-test. FSFI: Female Sexual Function Index.

## Data Availability

The datasets generated and/or analyzed during the current study are available from the corresponding author on reasonable request.

## Abbreviations

The following abbreviations are used in this manuscript:

MFP	Midline Rectovaginal Fascia Plication
FSFI	Female Sexual Function Index
VAS	Visual Analog Scale
